# The opportunities and challenges for nutritional intervention in childhood cancers

**DOI:** 10.3389/fnut.2023.1091067

**Published:** 2023-02-13

**Authors:** Kaiyue Wang, Tianyou Yang, Yubin Zhang, Xiang Gao, Ling Tao

**Affiliations:** ^1^Department of Nutrition and Food Hygiene, School of Public Health, Institute of Nutrition, Fudan University, Shanghai, China; ^2^Department of Pediatric Surgery, Guangzhou Women and Children’s Medical Center, Guangzhou Medical University, Guangzhou, China

**Keywords:** malnutrition, pediatric cancer, nutrient dependency, dietary modifications, precision nutrition

## Abstract

Diet dictates nutrient availability in the tumor microenvironment, thus affecting tumor metabolic activity and growth. Intrinsically, tumors develop unique metabolic features and are sensitive to environmental nutrient concentrations. Tumor-driven nutrient dependencies provide opportunities to control tumor growth by nutritional restriction or supplementation. This review summarized the existing data on nutrition and pediatric cancers after systematically searching articles up to 2023 from four databases (PubMed, Web of Science, Scopus, and Ovid MEDLINE). Epidemiological studies linked malnutrition with advanced disease stages and poor clinical outcomes in pediatric cancer patients. Experimental studies identified several nutrient dependencies (i.e., amino acids, lipids, vitamins, etc.) in major pediatric cancer types. Dietary modifications such as calorie restriction, ketogenic diet, and nutrient restriction/supplementation supported pediatric cancer treatment, but studies remain limited. Future research should expand epidemiological studies through data sharing and multi-institutional collaborations and continue to discover critical and novel nutrient dependencies to find optimal nutritional approaches for pediatric cancer patients.

## Introduction

Childhood cancers represent the leading cause of disease-related mortality in childhood (1). Major childhood cancer types include leukemias (i.e., acute lymphoblastic leukemia ALL, acute myeloid leukemia AML), lymphomas (i.e., Hodgkin lymphoma HL, non-Hodgkin lymphoma NHL), brain and spinal cord tumors (i.e., glioblastoma GBM, medulloblastoma MB), peripheral nervous system tumors (i.e., neuroblastoma NB), renal cancers (i.e., Wilms tumor WT), liver cancers (i.e., hepatoblastoma HB), eye cancers (i.e., retinoblastoma RB), bone cancers (i.e., osteosarcoma OS and Ewing sarcoma ES), and soft tissue sarcomas (i.e., rhabdomyosarcoma RMS). The mutational burden in most childhood cancers is substantially lower than that in adult cancers (2, 3). Instead, fusion oncoproteins and epigenetic dysregulations frequently occur in childhood cancers (1, 4). For example, the EWS-FLI1 fusion protein plays a central role in the pathogenesis of ES (4). Oncohistones and aberrant DNA methylations have been identified in pediatric brain tumors (5–7). In addition, copy number alterations such as *MYCN* amplification occurs in many pediatric cancer types, such as NB (8), MB (9), WT (10), RB (11), and RMS (12).

Malnutrition (undernutrition and overnutrition) problems are increasing worldwide (13), raising concerns about the relationship between nutrition and childhood cancers (Table 1). Nutrient deficiency and obesity at diagnosis are associated with poor clinical outcomes in childhood cancers (34, 45, 77–79). Maternal nutritional status also links to the risk of developing hematopoietic and solid childhood tumors (17–21). Overall, the current epidemiological studies are limited. This is partly due to a lower incidence of childhood cancers and little nutritional evaluation at diagnosis (80). On the other side of the coin, multi-omics technology corroborating with basic and translational cancer research sheds light on discovering new metabolic dependencies of pediatric cancers. Like adult cancers, aggressive pediatric tumors require specific lipids, amino acids, carbohydrates, vitamins, and minerals for survival (63, 81, 82). The most vulnerable metabolite is determined under a particular context of cancer and is associated with the tumor microenvironment (83). Finding critical nutrient dependencies for each cancer type will aid in developing optimal treatment regimens.

**Table 1:** Dietary associations and tumor-driven nutrient dependencies in pediatric cancers.

Pediatric cancer type	Pediatric cancer name	Associations between nutrient/diets and pediatric cancers	Tumor-driven nutrient dependencies
Leukemias	Acute lymphoblastic leukemia (ALL)	Protein-energy malnutrition (+) (14, 15) Magnesium and zinc deficiency (+) (16) Maternal obesity and diabetes (+) (17, 18) Maternal diet during pregnancy (vegetables, fruits, protein sources, and folate supplementation) (19–21)	Amino acids: glutamine (22), arginine (23), asparagine (24) Glycolysis and oxidative phosphorylation: glucose uptake (25) Lipids: mevalonate pathway (26) Vitamins: vitamin D (27)
	Acute myeloid leukemia (AML)	Maternal intake of dietary DNA topoisomerase II inhibitors (+) (28)	Amino acids: glutamine (29), arginine (23, 30) Glycolysis and oxidative phosphorylation: PDK1 pathway (31) Lipids: phosphatidylinositol, sphingolipids, free cholesterol, monounsaturated fatty acids (32, 33)
Lymphomas	Hodgkin lymphoma (HL)	Undernutrition (+) (34) Zinc deficiency (+) (16)	Not available
	Non-Hodgkin lymphoma (NHL)	Undernutrition (+) (34) Zinc deficiency (+) (16)	Glycolysis and oxidative phosphorylation: *HK* (35) Vitamins: vitamin D (36)
Brain and spinal cord cancers	Glioblastoma (GBM)	Not available	Amino acids: glutamine (37) Glycolysis and oxidative phosphorylation: *PDK1* (38) Lipids: ketone bodies (39)
	Medulloblastoma (MB)	Maternal diets during pregnancy (candy, chili peppers, and oil products +; fruits, −; yellow-orange vegetables, −) (40, 41)	Amino acids: glutamine (42) Glycolysis and oxidative phosphorylation: *HK* (43) Lipids: Smoothened-activating sterol lipids (i.e., cholesterol and 7-keto-cholesterol) (44)
Peripheral nervous system cancers	Neuroblastoma (NB)	Undernutrition (+) (45) Maternal folate fortification (−) (46)	Amino acids: glutamine (47, 48), SGOC metabolism (49, 50), polyamines (51–53) Glycolysis and oxidative phosphorylation: *HK* (54), *LDHA* (55), *GLUT1* (56), mitochondrial activity (57, 58) Lipids: fatty acid metabolism [*SLC27A2* (59), *FASN* and *SCD* (60, 61), *CPT1* (58)] Vitamins and Minerals: vitamin B12 (62), iron (63, 64)
Eye cancers	Retinoblastoma (RB)	Vitamin D (−) (36)	Glycolysis and oxidative phosphorylation: PDK1 pathway (65) Vitamins: vitamin D (36)
Renal cancers	Wilms tumor (WT)	Folate fortification (−) (66)	Glycolysis and oxidative phosphorylation: *GLUT1* (67), mitochondrial activity (68)
Liver cancers	Hepatoblastoma (HB)	Vitamin D deficiency (+) (36)	Glycolysis and oxidative phosphorylation*: LDHB* (69), *GLUT3* (69) Lipids: fatty acid metabolism (*SREBP-1c* (70)) Vitamins: vitamin D (36)
Bone cancers	Osteosarcoma (OS)	Vitamin D deficiency (+) (36)	Amino acids: SGOC metabolism Glycolysis and oxidative phosphorylation: *HK* (71) Lipids: lipid catabolism and hydroxylation (72) Vitamins: vitamin D (36)
	Ewing sarcoma (ES)	Zinc deficiency (+) (73)	Amino acids: SGOC metabolism (74) Glycolysis and oxidative phosphorylation: *LDHA* (75)
Soft tissue sarcomas	Rhabdomyosarcoma (RMS)	Not available	Lipids: fatty acid oxidation (76)

(+) positive association; (−) negative association.

Here, we summarize recent findings on the associations between nutrition and pediatric cancers, nutritional dependencies under different tumor contexts, and dietary approaches during pediatric cancer treatment. Much remains to be uncovered in pediatric cancers compared to adult cancers. Thus, we also discuss the current challenges and research gaps in the field and point to interesting future directions. The ultimate goal is finding optimal and precise nutritional strategies to improve patient survival and quality of life.

## The link between nutrition and childhood cancers

The State of Food Security and Nutrition in the World reported that 9.8 percent of the global population (768 million) were undernourished in 2021 (13). Infants and children are more susceptible populations due to the high demand for energy and essential nutrients, especially for cancer patients. In a prospective study of 1,787 newly diagnosed pediatric patients, 18% had moderate nutritional depletion, and 45–59% were severely depleted (34). Another study showed that 50% of children with stage IV NB (high-risk patients) were undernourished at diagnosis (45). Undernourished children abandoned therapy more frequently, resulting in inferior event-free survival (34). Protein-energy malnutrition, a specific undernutrition defined as an energy deficit due to a lack of macronutrients, is commonly seen in leukemias and solid tumors (14, 15). Apart from the macronutrients, deficiencies of micronutrients such as magnesium, zinc, selenium, vitamin D, and vitamin B12 were reported in pediatric cancer patients (Table 1) (16, 36, 73, 84). There is a lack of standard clinical practice guidelines for monitoring the nutritional status of children with cancer. Therefore, a systematic comparison of different evaluation methods and longitudinal nutritional assessment throughout diagnosis and treatment is urgently needed.

The number of overweight and obese individuals (85) and the cancer risk (16–18) have increased over the years. A recent study of 640 pediatric ALL patients found that 27% were overweight/obese, and 79% exceeded the dietary reference amount (79). Obese pediatric acute leukemia patients had a higher mortality risk than non-obese patients (77, 78). In recent years, the consumption of food-added sugars has also increased dramatically. High sugar consumption was associated with increased incidences of multiple adult malignancies, such as pancreatic and endometrial cancers (86, 87). However, whether sugar contributes to pediatric cancers is poorly understood.

Maternal obesity and diabetes also increase the risk of childhood cancers. Children born to mothers with a body mass index of ≥40 had a 57% higher leukemia risk (18). Maternal diabetes was associated with an increased risk of childhood cancers, particularly ALL, and medications reduced the risk of offspring childhood cancers (17). Additionally, maternal diets correlate with the risk of childhood cancers. Maternal consumption of vegetables and fruits before or during pregnancy was inversely associated with offspring ALL and AML incidence (19, 20, 28). In contrast, consuming flavonoid-rich foods may interfere with DNA topoisomerase II and increase the risk of AML (28). Maternal folate fortification correlated with a reduced risk of ALL, NB, and WT (21, 46, 66). Additionally, a maternal diet rich in yellow-orange vegetables, fresh fish, and grains decreased the risk of childhood brain tumors, whereas a maternal diet rich in cured meats, eggs/dairy, oil products, non-chocolate candy, and chili increased the risk (41).

The current epidemiological studies remain limited, particularly for rare diseases. Future efforts should increase subjects through data sharing and multi-institutional collaborations.

## Nutrient dependencies in childhood cancers

Metabolic reprogramming has emerged as an essential cancer hallmark (88). Mutation of tumor suppressors and activation of oncogenic signaling make tumor cells promote the synthesis and uptake of nutrients for survival (89), thus enhancing tumor dependency on certain nutrients (81) (Table 1).

### Amino acids

Amino acids are the building blocks of proteins. They also regulate the redox state and contribute to epigenetic and immune responses linked to tumorigenesis and metastasis (90). Therefore, tumors present a heightened amino acid dependence.

Glutamine, the most abundant amino acid in serum, is surprisingly depleted in developing cancers (91). Glutamine supported childhood AML and ALL survival and contributed to adipocyte-induced cell resistance to asparaginase (22, 29). Inhibition of glucose metabolism or Akt signaling also activated glutamine metabolism in GBM (37). In NB, the oncogenic driver MYCN promoted glutamine uptake and catabolism (47, 48). Similarly, high *MYC*-expressing atypical teratoid/rhabdoid tumors demonstrated higher glutamine metabolism activity compared to low *MYC*-expressing tumors (92). TAp73, which is frequently overexpressed in human tumors, sustained a subset of MB growth and proliferation by upregulating glutamine metabolism (42).

Another critical amino acid pathway is the serine-glycine-one-carbon (SGOC) metabolism. SGOC incorporates serine–glycine biosynthesis, one-carbon metabolism, and purine nucleotide biosynthesis in a positive feedback loop, generating diverse metabolites (93). In NB, high expression of an SGOC gene signature (49) or glycine decarboxylase (50), the enzyme which catalyzes glycine breakdown to produce one-carbon metabolism intermediate 5,10-methylene-tetrahydrofolate, was identified in *MYCN*-amplified patients and was associated with advanced disease stage and poor prognosis. In OS, the rate-limiting enzyme in serine biosynthesis 3-phosphoglycerate dehydrogenase is inversely correlated with patient survival (94). In ES, two methylenetetrahydrofolate dehydrogenase genes (*MTHFD2* and *MTHFD1L*) were upregulated by EWS-FLI1, and high expressions were linked with high-risk disease and poor survival (74).

Arginine is a semi-essential amino acid and an intermediate in many biological pathways, such as the urea cycle and tricarboxylic acid cycle (95). AML depends on arginine, as depletion of intracellular arginine (*via* a pegylated arginine deiminase ADI-PEG20) and extracellular arginine (*via* a pegylated human recombinant arginase BCT-100) decreased proliferation of AML (23, 30). Depletion of arginine in combination with chemotherapy cytarabine exerted greater efficacy compared to single therapy in AML and ALL. Still, resistance eventually occurred (30, 96), likely due to compensatory activation of endogenous production of arginine (23, 97). Therefore, additional metabolic dependencies in AML must be targeted.

Asparagine is a nonessential amino acid that facilitates glycoprotein synthesis and the uptake of extracellular amino acids such as arginine, histidine, and serine (98). Asparagine presents a potential nutrient dependency in leukemias as these tumors lack asparagine synthetase (24, 99). Deprivation of exogenous asparagine by asparaginase resulted in a remission rate of >90% in children with ALL (24).

Polyamines are active organic compounds with at least two amino groups (100). They can be synthesized by ornithine decarboxylase (*ODC1*), the rate-limiting enzyme in polyamine synthesis (100), and imported by transporters such as *SLC3A2* (53). Polyamines are frequently deregulated in cancer because they involve fundamental processes related to cell growth and survival (100). For example, putrescine, spermidine, and spermine levels were elevated in children with leukemias (101). MYCN directly increased polyamine synthesis in NB and promoted NB tumor growth by upregulating *ODC1* and *SLC3A2* (51–53).

### Glycolysis and oxidative phosphorylation

As discovered by Otto Warburg in the 1920s, cancer cells exhibit an increased dependence on glycolysis, preferentially catalyzing the conversion of glucose to lactate in the presence of oxygen (102). Despite a relatively low mutational burden, pediatric cancers exhibit aberrant expressions of key glycolytic enzymes, suggesting an increased dependence on glycolysis. Expression of hexokinase (*HK1/2*), which converts glucose to glucose-6-phosphate, was raised in several pediatric cancers and high *HK* expression predicted poor prognosis [i.e., metastatic NB (54), the SHH subtype of MB (43), diffuse large B-cell lymphoma (35) and OS (71)]. High expression of lactate dehydrogenase (*LDHA/B*) that converts pyruvate to lactate is linked to poor prognosis in NB, ES, and HB (55, 69, 75). Additionally, glucose transporter *GLUT1* was highly expressed in ALL (25), NB (56), and WT (67), and *GLUT3* upregulated in HB (69).

More recent work has demonstrated that mitochondrial respiration also plays a significant role in tumor growth and survival (103). WT and NB exhibited heterogeneity in mitochondrial phenotypes and energy metabolism (57, 58, 68). Stromal regions of WT showed reduced mtDNA copy number, whereas the epithelial and blastemal regions were normal (68). *MYCN*-amplified NB demonstrated higher mitochondrial activity than non-*MYCN-*amplified NB (57, 58). The pyruvate dehydrogenase kinase 1 (PDK1) phosphorylates pyruvate dehydrogenase and in turn, lowers its activity, which reduces the conversion of pyruvate to acetyl-CoA. PDK1 was activated in AML, GBM, and RB, and its inhibition blocked cell proliferation and restored sensitivity to chemotherapy (31, 38, 65).

### Lipids

Lipids are also critical for cancer cell proliferation by serving as membrane components, providing energy sources, maintaining redox homeostasis, and acting as signaling molecules (104). Several pediatric cancers heavily rely on lipids for survival. For example, fatty acid transport (*via* fatty acid transporter *SLC27A2*), biosynthesis (*via* fatty acid synthase *FASN* and stearoyl-CoA desaturase *SCD*), and oxidation (*via* carnitine palmitoyltransferase 1 *CPT1*) were activated in *MYCN*-amplified NB (58–61). Inhibition of fatty acid oxidation by malonyl-CoA decarboxylase inhibitor prohibited RMS growth, and knockdown of fatty acid metabolism regulator sterol regulatory element-binding protein-1c (*SREBP-1c*) suppressed HB, suggesting a dependency on lipid metabolism (70, 76). Lipids such as phosphatidylinositol, sphingolipids, free cholesterol, and monounsaturated fatty acids were increased in isocitrate dehydrogenases mutant AML cells (32). Moreover, AML blasts activated adipocyte lipolysis, thus allowing fatty acids to be transferred from adipocytes to blasts (33). A recent study identified the mevalonate pathway as a novel vulnerability in early T-cell ALL (26). Inhibition of 3-hydroxy-3-methylglutaryl-CoA reductase, the rate-limiting enzyme in the mevalonate pathway, significantly blocked T-cell ALL growth (26). Besides, smoothened-activating sterol lipids such as cholesterol and 7-keto-cholesterol sustained oncogenic Hedgehog signaling in MB (44).

### Vitamins and minerals

Besides the macronutrients, vitamins and minerals are essential as substrates and cofactors for critical metabolic processes (105). Pediatric cancer patients were disproportionately vitamin D deficient (27, 36, 82), suggesting sequestration of this vitamin by tumor cells. Vitamin B12 has also been identified as a pediatric cancer dependency, specifically in NB cells, such that B12 depletion induced cell-cycle arrest and neuronal differentiation (62).

Among minerals, iron serves as a significant dependency across pediatric cancers. Iron depletion by iron chelator deferoxamine or sodium ascorbate has demonstrated anti-proliferative activity in NB (63, 64). However, exogenous iron exposure induced ferroptosis in malignant brain tumors and NB (106–108), indicating that tight control of iron levels is required for cancer cell survival.

## Dietary modifications in pediatric cancers

Inhibitors used to disrupt active cancer metabolism have been extensively summarized in many reviews (81, 109–111). Nevertheless, a majority of drugs fail to enter clinical trials. Compensatory activation of other metabolic routes or uptake of the source metabolite reduces the anti-tumor effects (59, 112). Moreover, the specificity of the metabolic inhibitors to tumor cells and their potential toxicities remain a question. Therefore, supportive approaches such as dietary modifications should be considered during cancer treatment. Dietary composition determines nutrient availability in the microenvironment of cells in the body (113). Accumulating evidence suggests that dietary modifications, including calorie and nutrient restriction/supplementation, reprogram tumor metabolic activity and produce shifts in proliferation rate and drug sensitivity (114, 115). Herein, this review will focus on dietary modifications applied to pediatric cancers.

### Calorie restriction and ketogenic diet

Calorie restriction emphasizes a chronic 20–30% reduction of the standard calorie intake (116). This approach reduces tumor growth in several adult tumor models, including breast cancer, pancreatic cancer, and lymphoma (117–119). Interestingly, calorie restriction also inhibited tumor growth in neuroblast mouse xenograft models, although its molecular mechanism remains unknown (120). However, it remains a concern to use calorie restriction during childhood, given the risk of malnutrition and disrupted endocrine function (121).

Consequently, researchers have sought a safer approach that sustains overall calorie intake but modifies the diet composition. The ketogenic diet was introduced to meet such demand. Ketogenic diets have normal calorie, low-carbohydrate but high-fat content, leading to increased ketone bodies in plasma (122). The ketogenic diet showed anticancer effects in preclinical models of NB and GBM (39, 120, 123). Researchers further identified that a medium-chain fatty acid-containing ketogenic diet was more effective than a long-chain fatty acid-containing diet (120, 123). It remains uncertain whether ketogenic diet can be applied to all cancer types.

### Nutrient restriction

Germline mutations in the methionine synthase gene have been associated with childhood leukemia risk (124), and methionine depletion augmented the anticancer activities of chemotherapeutics against pediatric sarcoma cells *in vitro* (125). However, it remains unknown whether methionine dependence is a broader feature across pediatric cancers and whether this dependency can be effectively exploited against tumors in children. Besides methionine, serine deprivation has also been shown to induce oxidative stress and prohibit tumor growth in adult cancer models (126, 127). Given that NB and ES showed active serine metabolism (50, 74), it will be interesting to determine whether serine restriction is effective in treating those cancers.

Besides amino acids, restriction of vitamins can be used in tumors that rely on those vitamins for survival. Restriction of vitamin B9 (folate) and B12 (cobalamin) together with other methyl donors (methionine and choline) inhibited one-carbon metabolism and protected against adenoma development (128). Restriction of minerals like iron may selectively target cancer cells (63, 64). However, care must be taken to avoid restriction toxicity since pediatric cancer patients are frequently vitamin deficient already (82, 129).

### Nutrient supplementation

Omega-3 fatty acids exert anti-inflammatory and anti-tumor effects (130). Docosahexaenoic acid (DHA) and eicosapentaenoic acid (EPA) slowed MB tumor growth by alleviating the inflammatory tumor microenvironment (131). *MYCN*-amplified NB contained lower DHA levels than non-*MYCN*-amplified NB. DHA supplementation delayed the progression of NB in cell line-derived mouse xenograft models (132). However, another study using TH-*MYCN* transgenic NB model did not observe significant DHA effects (133). More studies and standard treatment strategies are needed to evaluate the efficacy of DHA supplementation in NB.

Folate and vitamin B12 deficiency was found in ALL patients who showed anemia on maintenance therapy (134). Supplementation of these deficient micronutrients significantly alleviated anemia. Supplementation of vitamin K2 and D3 improved bone mineral density in ALL patients during intensive steroid therapy (135). Further research is needed to comprehensively characterize micronutrient status before and after treatment and to precisely monitor the effects of micronutrient supplementation on patients’ health conditions.

Cachexia is a complex syndrome presenting with decreased food intake, weight loss, muscle and adipose tissue wasting, and hormonal/metabolic abnormalities (136). Muscle wasting is associated with reduced protein synthesis and increased protein degradation (137). Oral supplementation of a mixture of amino acids partially reversed cachexia in patients with advanced solid tumors (138). Similarly, a diet enriched in leucine or branch-chain amino acids stimulated muscle protein synthesis and alleviated cachexia (139, 140). However, most studies were conducted on adult cancer patients. Further investigations into the effects of cachexia on pediatric tumor development and strategies for its reversal through dietary modifications are urgently needed.

## Challenges for nutritional interventions and future directions

Studies on dietary modifications for adult cancer treatment have provided insights into pediatric cancer interventions. Nevertheless, there is still a long way to go. First, additional studies are needed to understand specific nutrient dependencies in different pediatric cancers and to evaluate the efficacies of various dietary interventions. Second, because dietary modifications induce systemic responses that impact both tumor and tumor microenvironment, a holistic understanding of how the nutrient restriction or supplementation affects tumor and tumor microenvironment such as anti-/pro-tumor immune response, stromal cell activity, angiogenesis, and whole-body homeostasis is highly demanded. Third, the duration and start time for nutritional therapy as well as the long-term toxicity from nutrient deprivation or supplementation should be evaluated. Fourth, it should be emphasized that no treatment works for all types of cancers. For example, histidine supplementation made leukemia xenografted tumors more sensitive to methotrexate (141), whereas histidine depletion in a Drosophila model selectively limited the growth of *MYC*-dependent neural tumors (142). Thus, specific tumor context needs to be mentioned when advertising nutritional therapies.

## Conclusion

**Figure 1:**
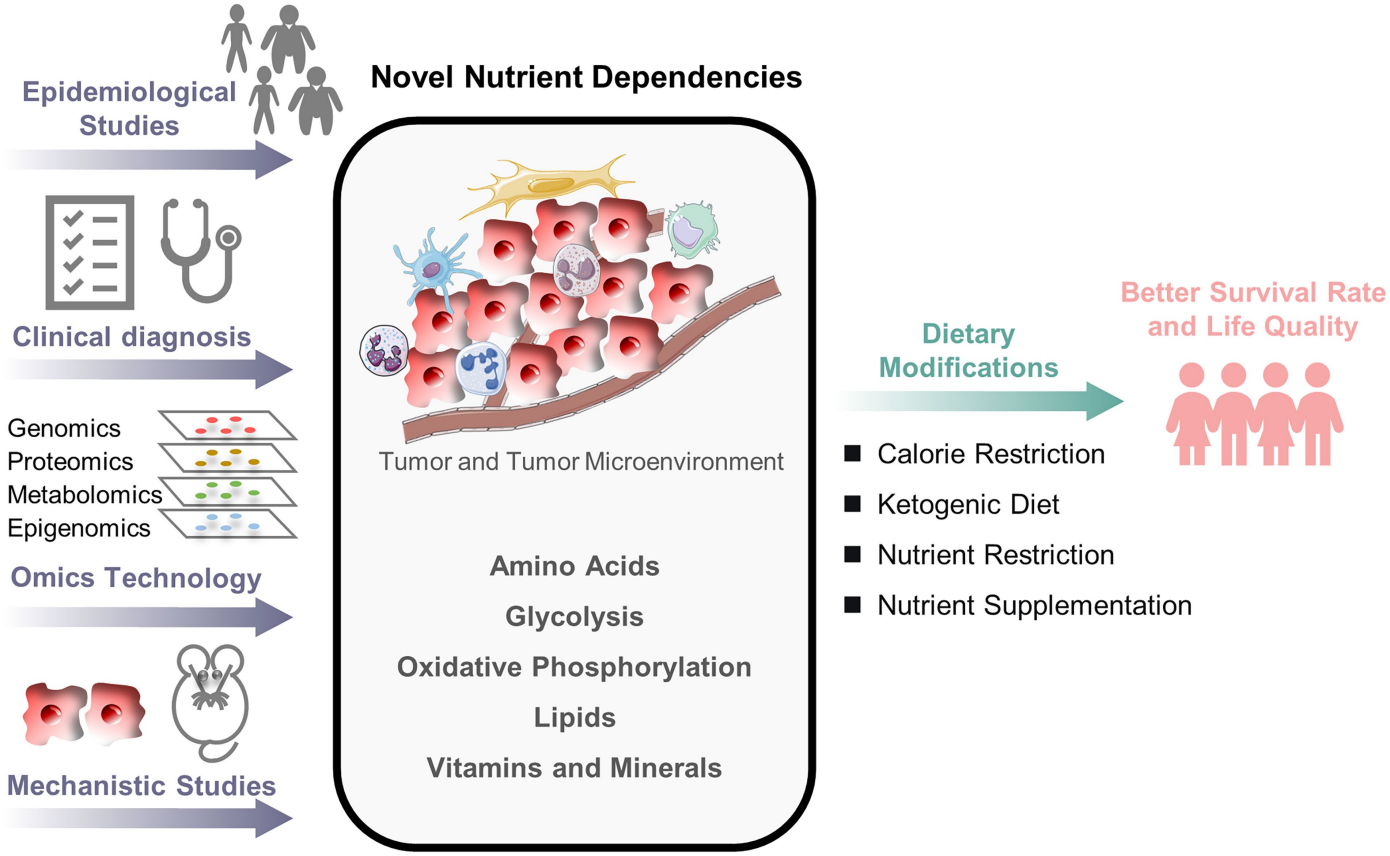
An outlook of nutritional research in pediatric cancers. The crosstalks between nutrition and pediatric cancers remain poorly understood. A combined approach of epidemiological investigation, clinical diagnosis, omics technology, and mechanistic study is necessary to understand better how nutrients alter cancer fate and to develop the optimal nutritional strategy for pediatric cancer patients.

Compared to adult cancers, the understanding of crosstalks between nutrition and pediatric cancers remains poor, thus providing tremendous opportunities for studying how nutrition can help prevent and treat childhood cancers (Figure 1). Large-scale epidemiological studies of pediatric cancers could uncover additional relationships between nutrients and pediatric cancers. Moreover, omics technology combined with mechanistic studies can reveal novel nutrient dependencies. This will further guide preclinical and clinical nutritional interventions to optimize therapeutic strategies. This process can be applied to populations, subgroups, and individuals. Each patient’s genetic information may guide the discovery of personalized nutrient dependencies and nutritional interventions. There is still much to learn, but additional studies will enlighten the future by overlapping nutrition and pediatric cancer fields.

## Author contributions

LT and KW wrote the manuscript and prepared the figure. LT and TY conceived and organized the structure of the review. KW, TY, YZ, XG, and LT reviewed the manuscript. LT revised the manuscript and supervised the entire writing process. All authors approved the final manuscript before submission and publication.

## Funding

This work was supported by Start-up Funding from Fudan University (to LT) and Sub-project of the Shanghai Local High-Level University Construction Program from Shanghai Municipal Education Commission (to LT).

## Conflict of interest

The authors declare that the research was conducted in the absence of any commercial or financial relationships that could be construed as a potential conflict of interest.

## Publisher’s note

All claims expressed in this article are solely those of the authors and do not necessarily represent those of their affiliated organizations, or those of the publisher, the editors and the reviewers. Any product that may be evaluated in this article, or claim that may be made by its manufacturer, is not guaranteed or endorsed by the publisher.

## Abbreviations

ALL: acute lymphoblastic leukemia
AML: acute myeloid leukemia
CPT1: carnitine palmitoyltransferase 1
DHA: docosahexaenoic acid
EPA: eicosapentaenoic acid
ES: Ewing sarcoma
FASN: fatty acid synthase
GBM: glioblastoma
GLUT: glucose transporter
HB: hepatoblastoma
HK: hexokinase
HL: Hodgkin lymphoma
LDHA: lactate dehydrogenase
MB: medulloblastoma
MTHFD: methylenetetrahydrofolate dehydrogenase
NB: neuroblastoma
NHL: non-Hodgkin lymphoma
ODC1: ornithine decarboxylase
OS: osteosarcoma
PDK: pyruvate dehydrogenase kinase
RB: retinoblastoma
RMS: rhabdomyosarcoma
SCD: stearoyl-CoA desaturase
SGOC: serine-glycine-one-carbon
SLC27A2: fatty acid transporter
SLC3A2: Polyamine transporter
SREBP-1c: sterol regulatory element-binding protein-1c
WT: Wilms tumor
